# Spindle cell lipoma of the anterior triangle of the neck: a rare entity

**DOI:** 10.1590/S1808-86942011000300021

**Published:** 2015-10-19

**Authors:** Smita Upadhyay, Arpit Sharma, Shashikant Mhashal, Jyoti P Dabholkar

**Affiliations:** 1 M.S. (senior resident); 2 M.S. (lecturer); 3 M.S. (lecturer); 4 M.S. (professor and head)

**Keywords:** immunohistochemistry, lipoma, neck

## INTRODUCTION

Solitary lipomas are the most common soft tissue tumor. Lipomas include a gamut of subtypes. Spindle cell lipoma (SCL) is a distinct entity accounting for about 1.5% of all lipomas^1^. The diagnosis of SCL is a histological one and careful study is required to differentiate it from the liposarcoma.

## CASE REPORT

A 48 year old male reported with a history of a left sided neck swelling since 2 years which was progressively increasing in size. A 4x4 cm swelling was present in the anterior triangle of the neck which was firm and mobile. ENT examination and investigations were unremarkable.

**Figure 1:**
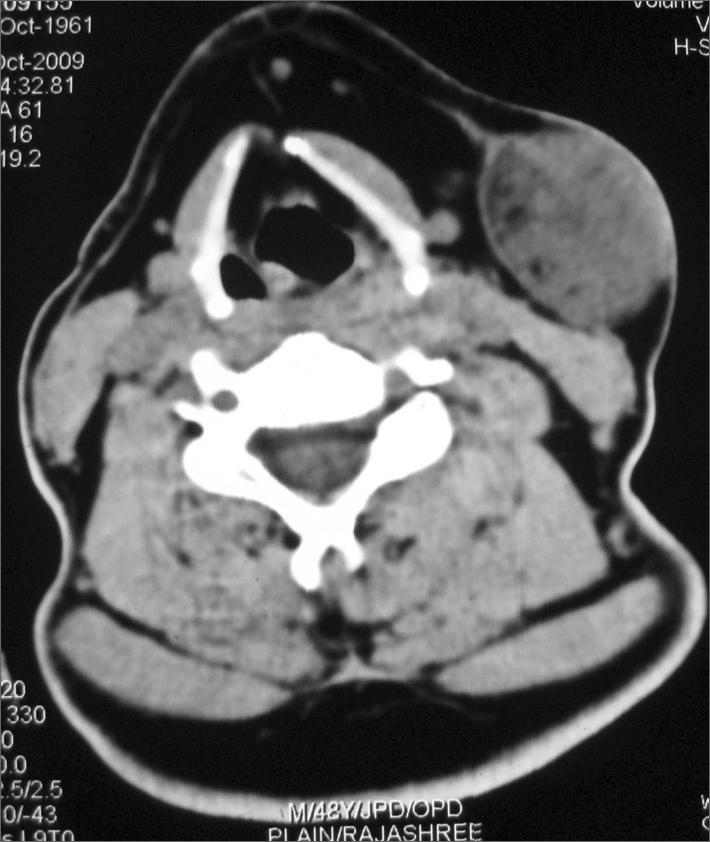
ct scan (axial) - ct scan showing a 3.2x4.2 cm soft tissue density with tiny densities of fat attenuation. lesion is seen displacing the platysma

Fine needle aspiration cytology showed loose arrangement of spindle cells in the myxoid matrix, suggestive of a soft tissue lesion. On computed tomography scan a 3.2x4.2 cm sized soft tissue density was seen in close proximity to the superficial lobe of the left parotid with tiny hypodensities of fat attenuation in the periphery. The lesion was extending anterior to the sternocleidomastoid muscle displacing the platysma. Post contrast showed a heterogenous enhancement. (Fig. 1)

Patient underwent surgical excision of the mass. The mass was subcutaneous well defined and encapsulated. The mass was excised completely.

On histopathological examination the tumor was composed predominantly of spindle cells which were uniform with an elongated nucleus with inconspicuous nucleloli. The cells were arranged in short parallel bundles. The background showed mucoid matrix mixed with ropey collagen fibre bundles. Mature adipocytes were also seen. On immunohistochemistry CD 34 antigen was positive and a diagnosis of spindle cell lipoma was made.

One year post operatively there is no evidence of a recurrence.

## DISCUSSION

Enzinger and Harvey first reported on SCL in 1975^2^. The condition predominantly involves the elderly males. Most common locations involve the posterior neck, shoulder and back however in our case the anterior triangle of the neck were involved.

The radiological appearance is not pathognomic. The variation in the ratio of fat and spindle cell causes the wide spectrum of imaging features. On T1 weighted MRI the lesion is isointense to the subcutaneous fat. T2 weighted MRI with fat suppression reveals a hypointense lipomatous component and a hyperintense spindle cell component which shows enhancement on contrast^3^.

Histologically SCL consists of a mixture of bland spindle cell and mature adipocytes. The matrix is composed of varying amounts of mucoid material and collagen. The spindle cells have scant cytoplasm, elongated nuclei and are arranged in short parallel arrays. The amount of adult fat is variable and lipoblasts are generally not seen^2^. Bundles of dense ropey collagen are present between the spindle cells. The spindle cells are CD34+ve and are S100-ve^4^.

Pleomorphic lipoma which is considered a highly pleomorphic variant was first described by Shmookler and Enzinger, as both of these have similar cytogenetics they are considered as a single entity^5^.

It is important to differentiate SCL from liposarcoma. Liposarcoma are characterized by more numerous spindle cells, presence of nuclear pleomorphism and MDM-2 amplification^1^. Myxoid stroma can sometimes be seen in SCL when it mimics myxoid liposarcomas. However the superficial location and the circumscribed lesion helps differentiate SCL from the latter. Also the vascular pattern, pleomorphism and mitotic activity should be assessed^6^. The other differential diagnosis includes dermatofibrosarcoma protuberans, mammary type solitary fibrous tumor and myofibroblastoma.

The treatment is surgical excision and recurrences are extremely rare even with incomplete excision.

## CONCLUSION

Spindle cell lipoma is an extremely rare variant of the common lipoma. The condition is benign and careful identification of the lesion is necessary to avoid misdiagnosing it as its malignant counterpart. Treatment involves surgical excision with virtually no recurrences.
